# Supplementary material for: A fault-intrusion-tolerant system and deadline-aware algorithm for scheduling scientific workflow in the cloud
Source: PeerJ Comput Sci. 2021 Nov 2;7:e747. doi: 10.7717/peerj-cs.747 (PMC8576554; doi:10.7717/peerj-cs.747)
Supplement: Supplemental Information 6 [file peerj-cs-07-747-s006.zip › Myproject1.1/docs/org/cloudbus/cloudsim/network/package-use.html]

Uses of Package org.cloudbus.cloudsim.network


JavaScript is disabled on your browser.


- Overview
- Package
- Class
- Use
- Tree
- Deprecated
- Index
- Help

- Prev
- Next

- Frames
- No Frames

- All Classes

# Uses of Package org.cloudbus.cloudsim.network

- Packages that use org.cloudbus.cloudsim.network

  | Package | Description |
  | --- | --- |
  |  |  |
  | --- | --- |
  | org.cloudbus.cloudsim |  |
  | org.cloudbus.cloudsim.network |  |
- Classes in org.cloudbus.cloudsim.network used by org.cloudbus.cloudsim

  Class and Description

  DelayMatrix\_Float

  This class represents an delay-topology storing every distance between connected nodes

  TopologicalGraph

  This class represents an graph containing nodes and edges, used for input with an network-layer
  Graphical-Output Restricions! EdgeColors: GraphicalProperties.getColorEdge NodeColors:
  GraphicalProperties.getColorNode
- Classes in org.cloudbus.cloudsim.network used by org.cloudbus.cloudsim.network

  Class and Description

  GraphReaderIF

  This interface abstracts an reader for different graph-file-formats

  TopologicalGraph

  This class represents an graph containing nodes and edges, used for input with an network-layer
  Graphical-Output Restricions! EdgeColors: GraphicalProperties.getColorEdge NodeColors:
  GraphicalProperties.getColorNode

  TopologicalLink

  This class represents an link (edge) from an graph

  TopologicalNode

  Just represents an topological network node retrieves its information from an
  topological-generated file (eg.

- Overview
- Package
- Class
- Use
- Tree
- Deprecated
- Index
- Help

- Prev
- Next

- Frames
- No Frames

- All Classes
